# The challenges of neural mind-reading paradigms

**DOI:** 10.3389/fnhum.2013.00306

**Published:** 2013-06-28

**Authors:** Oscar Vilarroya

**Affiliations:** Departament de Psiquiatria i Medicina Legal, Universitat Autonoma de Barcelona and Fundació IMIMBarcelona, Spain

**Keywords:** multivariate pattern analysis, neural encoding, neural decoding, neural representation, mind reading

## Abstract

Neural mind-reading studies, based on multivariate pattern analysis (MVPA) methods, are providing exciting new studies. Some of the results obtained with these paradigms have raised high expectations, such as the possibility of creating brain reading devices. However, such hopes are based on the assumptions that: (a) the BOLD signal is a marker of neural activity; (b) the BOLD pattern identified by a MVPA is a neurally sound pattern; (c) the MVPA's feature space is a good mapping of the neural representation of a stimulus, and (d) the pattern identified by a MVPA corresponds to a representation. I examine here the challenges that still have to be met before fully accepting such assumptions.

Neural mind-reading studies are booming and providing exciting new paradigms that map BOLD signals into stimuli features, and vice versa [see (Naselaris et al., 2011; Haxby, 2012; Serences and Saproo, 2012; Tong and Pratte, 2012); Mur et al. (2009) for recent reviews]. The breakthrough for neural mind-reading studies has been the development of multivariate pattern analysis (MVPA) tools applied to BOLD data. Researchers have used this set of tools with two main strategies: decoding and encoding. The decoding strategy attempts to correlate brain activity with co-occurring stimuli, behavior or cognitive activity. By contrast, the encoding strategy attempts to do exactly the opposite, namely, to predict activity in the brain evoked by co-occurring stimuli, behavior or cognitive activity. Both strategies can be pictured as a three space model: the stimuli input, the feature space, and the ROI's BOLD activity [see Figure 1, extracted from reference Naselaris et al. (2011)]. A visual stimulus, for instance, is characterized along axes that correspond to the luminance of each pixel, and a natural scene is represented by a single point in the input space. In such a feature space, each axis corresponds to a single feature, and each stimulus is represented by one point in that feature space. In general, the feature space provides labels that reflect different interpretations of the stimuli (e.g., inanimate versus animate), but they can also consist of a continuous representation, such as phase-invariant Gabor wavelets (Naselaris et al., 2009). Finally, the activity space represents the activation of all the voxels within an ROI: the axes correspond to the individual voxels, and ROI's activation pattern is identified by a unique point in the activity space. In general, the transformation from the input space into the feature space is a nonlinear mapping, whereas the transformation from the feature space to the particular BOLD activity, or vice versa, is a linear mapping. In this later case, the mapping involves training a linear classifier that allows mapping multi-voxel activation patterns onto specific stimulus labels.

**Figure 1:**
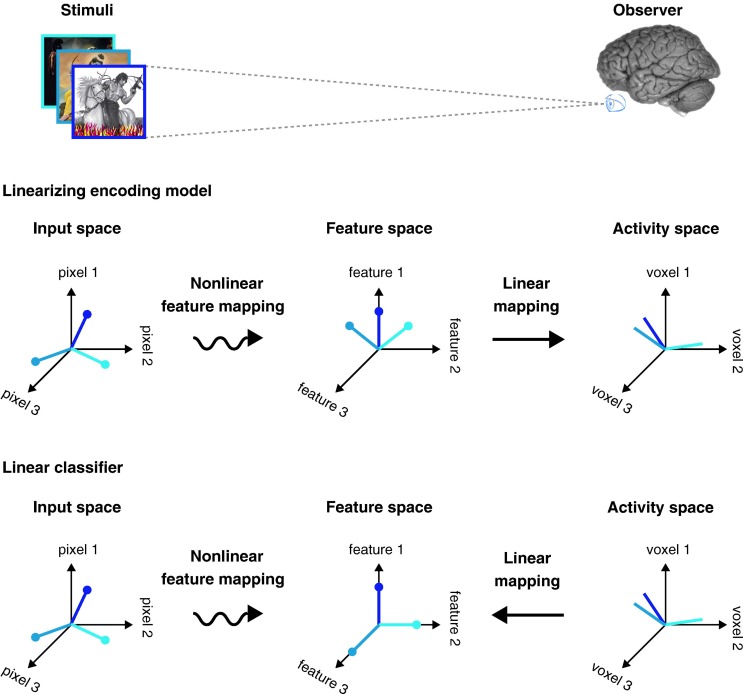
**Linearizing encoding and decoding models. Top:** The brain can be viewed as a system that maps stimuli onto brain activity in a nonlinear fashion. According to this perspective, a central task of systems and cognitive neuroscience is to discover the nonlinear mapping between input and activity. **Middle:** Linearizing encoding model. The relationship between encoding and decoding can be described in terms of a series of abstract spaces. In experiments using visual stimuli, the axes of the input space are the luminance of pixels and each point in the space (here different colors in the input space) represents a different image. Brain activity measured in each voxel is represented by an activity space. The axes of the activity space correspond to the activity of different voxels, and each point in the space represents a unique pattern of activity across voxels (different colors in the activity space). In between the input and activity spaces is a feature space. The mapping between the input space and the feature space is nonlinear and the mapping between the feature space and activity space is linear. **Bottom:** Linear classifier. Linear classifiers are simple decoding models that can also be described in terms of input, feature and activity spaces. However, the direction of the mapping between activity and feature space is reversed relative to the encoding model. Because the features are discrete all points in the feature space lie along the axes. Reprinted from Naselaris et al. (2011), with permission from Elsevier.

The MVPA approach has already yielded original and ambitious studies, including the prediction of neural signatures induced by visual stimuli (Mitchell et al., 2008), the quantification of the neural activity that can be attentionally biased (Reddy and Kanwisher, 2007), and the reconstruction of the image associated with a perceptual experience (Nishimoto et al., 2011). In this sense, the expectations about neural mind-reading paradigms are growing, and even reach visionary stances: “This [the study in question] is a critical step toward the creation of brain reading devices that can reconstruct dynamic perceptual experiences” (Nishimoto et al., 2011, p. 1645).

I will here assess two claims that underlie such expectations: (1) the claim that neural mind-reading paradigms can identify the neural pattern of neural representations; and (2) the claim that such paradigms can read out the contents of neural patterns. Let us examine each in turn.

## Neural signature's identification

The claim that MVPA-based studies can identify the neural pattern of neural representations is based on two assumptions: (a) the assumption that the BOLD signal is a marker of neural activity, and (b) the assumption that the BOLD pattern identified by MVPA is a neurally sound pattern.

Concerning (a), the fact is that relationships between changes in neural activity, the BOLD signal, and the cognitive state of the individual are still a matter of controversy. In principle, the magnitude of the BOLD signal is associated to the magnitude of underlying neural activation. This has led to the widespread consideration that there is a linear association between BOLD response and the implication of specific neural structures in cognitive processing. However, the BOLD signal actually measures blood oxygenation, which is an indirect measure of neural activity, and the relationship between cognitive functionality and the BOLD signal has not yet been resolved. Many aspects of the neural activity (e.g., synaptic activity, spiking activity, glial metabolic activity) contribute to the BOLD signal, and the functional implication of neurons underlying a BOLD signal is quite diverse (Logothetis and Wandell, 2004; Ekstrom, 2010; Gardner, 2010). Therefore, much more precise information about the relationship between the BOLD signal, neural activity and cognitive processing is needed before we rely on the BOLD-data analysis to understand the neural activity tapped with fMRI.

Assumption (b) holds that the BOLD pattern identified by MVPA is a neurally sound pattern. There are, however, still many questions to resolve before we can fully embrace this assumption. To begin with, how and why MVPA seems to be able to identify basic stimulus features in neural populations is still unclear. MVPA is thought to rely on the fact that each voxel contains different feature-selective neuronal populations. Univariate methods cannot discriminate the feature selectivity of such different populations, because they take the voxel as a whole. MVPA takes into account the pattern of activation across multiple voxels, and thus it seems to be able to discriminate among neuronal populations' feature selectivity [see for a review Haynes and Rees (2006); Mur et al. (2009)]. However, this has still to be proven (Freeman et al., 2011).

Secondly, different MVPA classifiers use different means in extracting information from the data (i.e., different classifiers encode feature covariance differently), and it is not clear yet how different types of classifiers affect MVPA performance. The variability in performance between classifiers with different learning strategies can provide different interpretations on how neurons are organized to encode stimuli features (Misaki et al., 2010; Yang et al., 2012).

Thirdly, depending on the approach, MVPA can identify local or non-local BOLD patterns. However, we still do not know whether neural representations are local or non-local, and hence how to interpret local or non-local patterns. Thus, patterns identified by MVPA might be an efficient classifying strategy, but cognitively misleading (Tong and Pratte, 2012). Note that the concern here is not about the BOLD signal itself; rather, it is about the fact that we lack independent evidence pointing to whether neural representations are local or non-local, and thus whether what we identify through MVPA is a part of a neural representation or a network of neural representations. In this sense, lesion studies, and other neuropsychological methods, would be independent evidence in support for either a local or non-local neural-representation hypothesis. Historically, neuropsychology has been providing strong evidence for local neural representations. However, some neuropsychological theorists have long since raised concerns about localizationism (Shallice, 1988; Patterson and Plaut, 2009), and recent studies on, for example, multisensory integration, also raise concerns about local neural representations (e.g., Shams et al., 2011).

Finally, the temporal resolution of the BOLD signal is poor. In the window of time that a BOLD signal is characterized, many relevant neural activities implicating many different neural representations might have taken place without being able to be detected by MVPA classifiers. Likewise, it is still not clear how changes in the BOLD signal during time are to be correlated with neural activity. Thus, even if in some cases it has been able to decode the dynamic experience of an individual from specific BOLD patterns (Nishimoto et al., 2011), we lack evidence to discern whether such a decoding is a classifier's artifact, or a genuine mapping.

In sum, there are still some neurophysiological and technical aspects of MVPA to be resolved. The challenges do not seem nevertheless insurmountable, and one can expect that they will eventually be met.

## Reading out neural contents

The claim that neural mind reading paradigms can read out the contents of the signature is also based on two assumptions: (c) the assumption that MVPA's feature space is a good mapping of the neural representation of a stimulus, and (d) the assumption that what MVPA identifies is a *representation* of a stimulus.

Regarding (c), the extraordinary results obtained by building ingenious and useful feature spaces should not neglect the fact that MVPA's linearizing feature spaces reflect a priori hypotheses about the stimuli features that might be represented. Indeed, the mapping is not between a set of stimuli and brain activity, but between an interpretation of the stimuli and an interpretation of brain activity. Hence, any feature selection must be very well supported. Even in some domains with a robust previous background, such as primary visual processing, the studies might not be in safe grounds. For example, classifiers may be able to distinguish face and furniture stimuli based on activation patterns in V1. However, without the a-priori knowledge that this region is known not to have category-preference, we can easily indulge in the fallacy of inferring the engagement of specific cognitive processes (categorization in V1) from patterns of activation (Yang et al., 2012).

In other domains, in which high-level processing is implicated, the decision about the feature space becomes much more speculative. In a neurosemantic study (Just et al., 2010), the authors assert that they identify a set of semantic features underlying the neural representation of concrete nouns, which are then used to identify “simple thoughts through their fMRI patterns” (Just et al., 2010, in the abstract). In the study, a factor analysis of the data provides a reduced number of factors that cluster the words comprising the stimuli set. The researchers assume these factors represent semantic dimensions and label them according to their best interpretation. However, showing that the identified factors are useful for classification and prediction only means the factors in question are good classifiers and predictors. It does not show that they constitute the word's semantic contents. The factors might reflect semantic dimensions that are not part of the neurosemantic representation of the word at issue, but just activated by it, as they could be markers of, for example, the word's semantic field or hypernymy. Furthermore, it is not at all clear that the factors actually reflect semantic dimensions at all. Some of the neural findings of the study overlap with findings from other fMRI studies in which brain areas and activations are related to other aspects that are not specifically semantic (such as motor planning). Thus, the factors might be simply good classifiers by the word's non-semantic implications, not for their semantic nature. Finally, it is not at all clear that the task activates the semantic representation of the word. The assumption that thinking about the properties of the object the word refers to specifically activates the word's representation is a very long shot. Among other challenges that could be advanced, the authors adopt a referential approach to semantics, they overlook that the properties of the object should activate the representations of the *properties* in themselves, and they assume without argument that asking to think of all properties of an object at the same time has the neural counterpart of effectively processing all the properties at the same time. Thus, it is far from clear that the semantic dimensions underlying the word meaning may appear in the task described in the study.

In sum, MVPA provides an inference to the best classification, but the best classification does not necessarily assure its relevance for a given neural pattern. Stimuli may differ in many features, and each one can yield differences in the BOLD signal. MVPA classifiers could pick up one feature, while the relevant cognitive features could be something else. MVPA can indeed classify stimuli that differ in ways that are irrelevant for cognitive classification, but which nevertheless create patterns in the BOLD signal. Furthermore, MVPA can even make up irrelevant classifications. For example, Hung and collaborators (Hung et al., 2005) were able to successfully classify the neural patterns for different stimuli that macaques, whose neural patterns were classified, were not able to distinguish (Anderson and Oates, 2010). As Logothetis put it, “voxels selective to two different stimuli attributes could be potentially detected by modern classifiers, yet the existence of two types of patterns does not necessarily imply the existence of two different types of neural populations” (Logothetis, 2008, p. 871).

Finally, assumption (d) states that what MVPA identifies is a *representation*. This is highly speculative. For one thing, it is not clear what is meant by “representation” in neural mind-reading studies. There are two main interpretations of the notion “representation.” One use can be identified with the notion of “process.” In a great deal of neural mind-reading studies, one can substitute the words “representation,” and “represented” with “process,” “processing,” or “processed,” and the meaning will not suffer: “Some regions of the human brain *represent* particular types of visually presented information in an anatomically segregated way. For example, the fusiform face area (FFA) is a region in the human ventral visual stream that responds more strongly to faces than to any other object category” (Haynes and Rees, 2006, p. 24, the italics are mine). In such cases, *represent* and *process* can be used interchangeably. However, there is also a strong use of the word: a neural representation might refer to some neural state or process containing information about *what* makes a thing *that* thing. In other words, if we were able to extract the information from such a state or process, we would be able to assess whether the owner had the knowledge of the thing or not. This seems to be the intended meaning in, for example, statements such as: “encoding models provide an explicit, quantitative description of how information is represented in the activity of individual voxels” (Naselaris et al., 2011, p.401).

The problem is that the neural implementation of a representation in this sense is still a mystery: we still do not know what it means for a nervous system *to represent* something, in the sense of “containing information of what makes a thing that thing.” The fact is that we lack a clear view on *what* makes a pattern a representation, and *what* is re-presented. The last serious discussions of these issues in a neuroscientific journal date back quite a while (Markman and Dietrich, 2000; Edelman, 2002; Wood and Grafman, 2003).

However, even if we do not know what it means for a neural system to represent something, we still can provide evidence supporting that something is in fact a representation. We can do so by either providing hard or operational evidence. Hard evidence consists of identifying the constitutive properties of a representation. Among other things, one may prove that a certain neural pattern contains some sort of structured information about what makes that pattern *the* representation of the element in question. Another relevant line of evidence is to show that the pattern is recruited in any inferential process whatsoever, because it contains information about the object that is relevant for the processing. Constitutive evidence may also be identifying the signature's form, and assess whether it is reproducible in other systems, with the same properties, and whether it is present in that form in other systems, with the same properties.

So far, none of this evidence has been provided by neural-mind reading paradigms. Moreover, at the moment, with the available methods in neuroscience, it is difficult to see how such constitutive properties of representations can be identified. Fortunately, identifying operational properties is a very good second option. Some MVPA studies already provide this sort of evidence and, hopefully, future studies will enlarge such operationally-based evidence.

One of such operational property requires showing that the neural signature *generalizes* to all cases involving a particular stimulus. Indeed, whatever counts as a neural representation, it must be able to identify the presence of the represented object in any of its instances, i.e., *always* that the object is implicated. Once we consider that a particular neural process or state is the neural representation of, say, “rabbit,” such a neural state should be present in all the instances of “rabbit.” Thus, operational evidence of the signature's ability to generalize would be to show that the signature is active in all the inferential processes in which the object is present, and preferably too in different cognitive modalities, such as in language, imagination and the like. Up to the moment, though, this is the least explored property of neural patterns in extant MVPA studies.

More studied operational evidence of representation is showing that the pattern is *detached* and *independent* from the thing it represents. Detached implies that the representation signature must be identified without the element it is about being present. Independent implies that the neural representation can function without the presence of the represented element, and has the same role with or without the represented element's presence. Indeed, representations allow individuals to distance themselves from the objects they represent (from the “here and now”) in thinking, planning or imagining. In this sense, evidence for detachment and independence would be showing that the signature is present, without its represented element, in processes like recall, imagination and thinking. Furthermore, it would also be relevant to show that the signature is induced by different cognitive modalities, such as language. In contrast with the lack of evidence for generalization, there are already promising studies in showing detachment and independence of BOLD patterns (Manning et al., 2012).

Finally, operational evidence for representation would be to show that the pattern is *stable* in time. In principle, a representation -the information about what makes a thing that thing- should not change, and nor should its neural pattern (Druckmann and Chklovskii, 2012). All things being equal, the particular information of “what makes a thing that thing” will always be the necessary and the sufficient information to count as the representation of “the thing,” and as such, it should be stable. Therefore, if we can attest that the neural pattern is stable, then we have good operational evidence that we are identifying the neural correlate of the representation. However, it is important to note that assuming BOLD signal stability as operational evidence does not imply that BOLD signal stability is required to identify a neural representation. Stability is a necessary property of neural representations, not of its BOLD signal correlate; it very well could be that eventually we might discover that BOLD signals cannot be stable for some particular reason, and thus stability as operational evidence could be ruled out. For example, the possibility of multiple realization in terms of different temporal representations could imply a relevant challenge to the stability requirement. Nonetheless, for the time being, BOLD signal stability can still be interpreted as the correlate of representation stability. At the moment, though, stability has not been used by extant neural mind-reading paradigms to identify neural representations. For one thing, there are technical factors interfering with the reliability of reading BOLD signals, which make it difficult to identify the same signal even for a within-subject repeated experimental session (Bennett and Miller, 2010). Additionally, BOLD responses change over time due to a great number of factors, not specifically related to the experimental context (Fliessbach et al., 2010). Therefore, studies specifically investigating the reliability and reproducibility of the BOLD signal will be required to help in establishing the requirements to consider BOLD patterns stable.

In sum, although providing constitutive evidence of representations is extremely difficult for extant neuroscientific methodology, providing evidence about operational properties of neural representations can allow a better grounding, and a larger basis, for the assumption that neural-mind reading paradigms are really reading out the contents of representations.

## Conclusion

The path that neural-mind reading paradigms have opened is promising, but there is a long way to go yet. As extraordinary as their results are, neural mind-reading paradigms do not yet provide sufficient evidence to grant their neural reading-out capacities. New studies and approaches are needed to reach a better understanding of the underlying neurophysiology of MVPA paradigms, a better modeling of feature spaces, and a better control of experimental paradigms. These points are hardly trivial. The design and interpretation of a great deal of studies are grounded in extant neural mind-reading studies, and the consequences they extract from previous studies are of great importance for future research. Meeting such challenges may allow present visionary statements to become a reality.

### Conflict of interest statement

The author declares that the research was conducted in the absence of any commercial or financial relationships that could be construed as a potential conflict of interest.
